# Gene Sampling Strategies for Multi-Locus Population Estimates of Genetic Diversity (*θ*)

**DOI:** 10.1371/journal.pone.0000160

**Published:** 2007-01-17

**Authors:** Matthew D. Carling, Robb T. Brumfield

**Affiliations:** 1 Museum of Natural Science, Louisiana State University, Baton Rouge, Louisiana, United States of America; 2 Department of Biological Sciences, Louisiana State University, Baton Rouge, Louisiana, United States of America; University of Cape Town, South Africa

## Abstract

**Background:**

Theoretical work suggests that data from multiple nuclear loci provide better estimates of population genetic parameters than do single loci, but just how many loci are needed and how much sequence is required from each has been little explored.

**Methodology/Principle Findings:**

To investigate how much data is required to estimate the population genetic parameter *θ* (4*N_e_μ*) accurately under ideal circumstances, we simulated datasets of DNA sequences under three values of *θ* per site (0.1, 0.01, 0.001), varying in both the total number of base pairs sequenced per individual and the number of equal-length loci. From these datasets we estimated *θ* using the maximum likelihood coalescent framework implemented in the computer program Migrate. Our results corroborated the theoretical expectation that increasing the number of loci impacted the accuracy of the estimate more than increasing the sequence length at single loci. However, when the value of *θ* was low (0.001), the per-locus sequence length was also important for estimating *θ* accurately, something that has not been emphasized in previous work.

**Conclusions/Significance:**

Accurate estimation of *θ* required data from at least 25 independently evolving loci. Beyond this, there was little added benefit in terms of decreasing the squared coefficient of variation of the coalescent estimates relative to the extra effort required to sample more loci.

## Introduction

Coalescent theory, placed in a maximum-likelihood framework, allows researchers to explicitly test hypotheses concerning the processes that shape patterns of genetic variation in natural populations [1]. Catalyzed by automated sequencing technologies as well as the availability of universal PCR primers (a direct benefit of genome projects), population genetic studies of natural populations have recently experienced a large-scale shift from single-locus investigations of cytoplasmic markers (e.g. mitochondrial, chloroplast) to multi-locus studies of autosomal and sex-linked loci. Although single-locus cytoplasmic gene trees are expected to outperform nuclear gene trees in some instances [2], [3], the statistical confidence of the inferences drawn from such trees is limited [4]–[6]. Consequently, the recombining histories of multiple nuclear loci are being used increasingly in empirical estimates of population genetic parameters [7]–[9].

It is well understood that coalescent estimates of population genetic parameters (e.g. effective population size, recombination rates) are often estimated with large errors when calculated from single-locus datasets [10], [11]. Simulation studies have demonstrated that the error rates associated with such parameter estimations can be reduced by analyzing data from multiple independent loci [12], [13]. Statistically, adding additional loci corresponds to gaining independent replicates of the underlying evolutionary processes [14]. Additionally, in some situations, greater precision of a population genetic parameter estimate can be achieved by sampling both shared polymorphisms and fixed differences between two populations, which can only be observed by sampling loci that have different genealogies [e.g. 13]. Multiple loci are also required in some methods for estimating the effective population size of ancestral populations [15], [16].

With the development of analytical methods for multiple-locus parameter estimation and the relative ease of collecting large amounts of sequence data, researchers with limited resources are concerned increasingly with how much data is required to estimate the parameters of interest accurately [17]. If more loci are better, as previous work has alluded to [12], [18], how many loci are needed and how many base pairs from each locus? Until an analytical solution to this question is developed, simulated data can provide insights into whether, given a fixed amount of sequencing effort, it is preferable to increase the number of loci or the sequence length of individual loci. Theory suggests that the number of independently segregating loci is crucial to the accurate estimation of the population genetic parameter θ (4*N_e_*μ) [14], [17], which is usually interpreted as a scaled measure of the neutral mutation rate per site or as the proportion of polymorphic sites in a population [19].

Our primary motivation was to investigate the accuracy and precision of estimates of θ, using a coalescent-based analytical method, across simulated datasets which vary in both the number of loci and the total number of base pairs sequenced per individual (e.g. 10 kb sequence/individual at one locus or distributed evenly among 10 loci). Whereas previous work in this area has focused primarily on making theoretical predictions of optimal sampling strategies [14], [17], our work uses extensive data simulations, over a broad range of both number of loci sampled and total sequence length sampled, to explore the validity of those predictions.

## Materials and methods

### Simulated Data

For three different per-site values of θ (0.1, 0.01, 0.001), we simulated DNA sequence data using Treevolve version 1.3 [20] according to scenarios that varied in both total amount of sequence per individual, as well as in the number of loci. Treevolve simulates DNA sequence evolution under a coalescent model. Although Treevolve can simulate sequences under a variety of population dynamic models, our data were simulated under a simple model with no selection, no intra-locus recombination, no population subdivision, and no migration. A population sample size of 10, which corresponds to phased nuclear haplotype data from five diploid individuals, was used in all simulations. We adjusted the Treevolve input parameters as follows: sequence length (varied according to scenario, see Table 1); mutation rate (1×10^−6^ for θ = 0.1; 1×10^−7^ for θ = 0.01, 0.001); number of loci sampled per individual (varied according to scenario, see Table 1); ploidy (haploid); generation time/variance in offspring number (1.0, corresponds to Wright-Fisher reproduction of non-overlapping generations and sampling with replacement); length of period (1×10^12^ upper limit on coalescent time, our value ensures all simulated sequences coalesce); population size (25000 for θ = 0.1, 0.01; 2500 for θ = 0.001); subdivision (1, corresponds to no population subdivision); and recombination (0.0). Sequences were simulated under the Felsenstein '84 [21] model of sequence evolution, with a transition/transversion ratio of 2.0 to model a moderate transition bias.

**Table 1:**
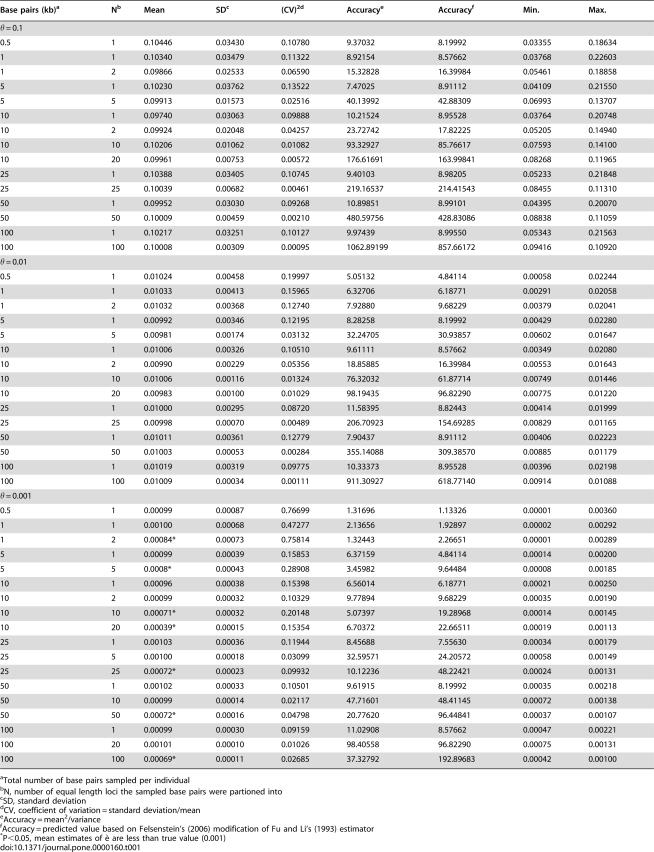
Simulation conditions and summary statistics for *è* calculations.

Base pairs (kb)^a^	N^b^	Mean	SD^c^	(CV)^2^ d	Accuracy^e^	Accuracy^f^	Min.	Max.
θ = 0.1								
0.5	1	0.10446	0.03430	0.10780	9.37032	8.19992	0.03355	0.18634
1	1	0.10340	0.03479	0.11322	8.92154	8.57662	0.03768	0.22603
1	2	0.09866	0.02533	0.06590	15.32828	16.39984	0.05461	0.18858
5	1	0.10230	0.03762	0.13522	7.47025	8.91112	0.04109	0.21550
5	5	0.09913	0.01573	0.02516	40.13992	42.88309	0.06993	0.13707
10	1	0.09740	0.03063	0.09888	10.21524	8.95528	0.03764	0.20748
10	2	0.09924	0.02048	0.04257	23.72742	17.82225	0.05205	0.14940
10	10	0.10206	0.01062	0.01082	93.32927	85.76617	0.07593	0.14100
10	20	0.09961	0.00753	0.00572	176.61691	163.99841	0.08268	0.11965
25	1	0.10388	0.03405	0.10745	9.40103	8.98205	0.05233	0.21848
25	25	0.10039	0.00682	0.00461	219.16537	214.41543	0.08455	0.11310
50	1	0.09952	0.03030	0.09268	10.89851	8.99101	0.04395	0.20070
50	50	0.10009	0.00459	0.00210	480.59756	428.83086	0.08838	0.11059
100	1	0.10217	0.03251	0.10127	9.97439	8.99550	0.05343	0.21563
100	100	0.10008	0.00309	0.00095	1062.89199	857.66172	0.09416	0.10920
θ = 0.01								
0.5	1	0.01024	0.00458	0.19997	5.05132	4.84114	0.00058	0.02244
1	1	0.01033	0.00413	0.15965	6.32706	6.18771	0.00291	0.02058
1	2	0.01032	0.00368	0.12740	7.92880	9.68229	0.00379	0.02041
5	1	0.00992	0.00346	0.12195	8.28258	8.19992	0.00429	0.02280
5	5	0.00981	0.00174	0.03132	32.24705	30.93857	0.00602	0.01647
10	1	0.01006	0.00326	0.10510	9.61111	8.57662	0.00349	0.02080
10	2	0.00990	0.00229	0.05356	18.85885	16.39984	0.00553	0.01643
10	10	0.01006	0.00116	0.01324	76.32032	61.87714	0.00749	0.01446
10	20	0.00983	0.00100	0.01029	98.19435	96.82290	0.00775	0.01220
25	1	0.01000	0.00295	0.08720	11.58395	8.82443	0.00414	0.01999
25	25	0.00998	0.00070	0.00489	206.70923	154.69285	0.00829	0.01165
50	1	0.01011	0.00361	0.12779	7.90437	8.91112	0.00406	0.02223
50	50	0.01003	0.00053	0.00284	355.14088	309.38570	0.00885	0.01179
100	1	0.01019	0.00319	0.09775	10.33373	8.95528	0.00396	0.02198
100	100	0.01009	0.00034	0.00111	911.30927	618.77140	0.00914	0.01088
θ = 0.001								
0.5	1	0.00099	0.00087	0.76699	1.31696	1.13326	0.00001	0.00360
1	1	0.00100	0.00068	0.47277	2.13656	1.92897	0.00002	0.00292
1	2	0.00084*	0.00073	0.75814	1.32443	2.26651	0.00001	0.00289
5	1	0.00099	0.00039	0.15853	6.37159	4.84114	0.00014	0.00200
5	5	0.0008*	0.00043	0.28908	3.45982	9.64484	0.00008	0.00185
10	1	0.00096	0.00038	0.15398	6.56014	6.18771	0.00021	0.00250
10	2	0.00099	0.00032	0.10329	9.77894	9.68229	0.00035	0.00190
10	10	0.00071*	0.00032	0.20148	5.07397	19.28968	0.00014	0.00145
10	20	0.00039*	0.00015	0.15354	6.70372	22.66511	0.00019	0.00113
25	1	0.00103	0.00036	0.11944	8.45688	7.55630	0.00034	0.00179
25	5	0.00100	0.00018	0.03099	32.59571	24.20572	0.00058	0.00149
25	25	0.00072*	0.00023	0.09932	10.12236	48.22421	0.00024	0.00131
50	1	0.00102	0.00033	0.10501	9.61915	8.19992	0.00035	0.00218
50	10	0.00099	0.00014	0.02117	47.71601	48.41145	0.00072	0.00138
50	50	0.00072*	0.00016	0.04798	20.77620	96.44841	0.00037	0.00107
100	1	0.00099	0.00030	0.09159	11.02908	8.57662	0.00047	0.00221
100	20	0.00101	0.00010	0.01026	98.40558	96.82290	0.00075	0.00131
100	100	0.00069*	0.00011	0.02685	37.32792	192.89683	0.00042	0.00100

a Total number of base pairs sampled per individual

b N, number of equal length loci the sampled base pairs were partioned into

c SD, standard deviation

d CV, coefficient of variation = standard deviation/mean

e Accuracy = mean^2^/variance

f Accuracy = predicted value based on Felsenstein's (2006) modification of Fu and Li's (1993) estimator

* *P*<0.05, mean estimates of *è* are less than true value (0.001)

By manipulating the user-defined mutation rate and population size, we were able to simulate sequences under known values of θ. With θ = 0.1 (μ = 1×10^−6^, *N_e_* = 25000), we first simulated 100 replicate datasets, each containing 10 single-locus DNA sequences 0.5 kb in length. Keeping θ = 0.1, we then simulated 100 more datasets that differed from the first datasets only in the length of each DNA sequence, which was increased to 1 kb. Next, 100 datasets were generated that contained DNA sequences from two unlinked loci, each 0.5 kb in length, sampled from 10 individuals. In these datasets the amount of DNA sequence per individual was equal to the previous datasets (1 kb), but was partitioned into two independent (i.e. no intra-genic recombination, but free inter-genic recombination) loci instead of being sampled from a single locus. In this way, we generated 100 replicates of datasets which varied in total DNA sequence length per individual from 0.5 kb to 100 kb, and the number of equal-length loci from which the sequences were sampled: 1 to 100 (15 total datasets, Table 1). We repeated the simulations with θ = 0.01 (μ = 1×10^−7^, *N_e_* = 25000; Table 1). The same 15 scenarios, plus three additional ones (25 kb per individual divided among five equal length loci, 50 kb among 10 loci, 100 kb among 20 loci), were simulated with θ = 0.001 (μ = 1×10^−7^, *N_e_* = 2500; Table 1). Output files were manipulated into the input format required by Migrate (see below) using a Perl script we wrote. We chose the values of θ to span the extremes of variation observed in natural populations [22]–[25]. Simulated datasets and the Perl scripts are available from the corresponding author.

### Calculations and Analyses

A number of software packages and analytical methods are available for estimating θ, such as such as SITES [13], Arlequin [26], GeneTree [27], and Beast [28]. Because methods that incorporate phylogenetic structure into the parameter estimation procedure have been demonstrated to have less bias [1], [29], [30], we calculated θ using the coalescent framework implemented in Migrate version 1.7.5 [31], but our results should be applicable to other methods of θ estimation. Using Markov chain Monte Carlo (MCMC) methods to approximate the likelihood distribution, Migrate calculates maximum likelihood estimates of population parameters under a coalescent framework. After discarding the first 10000 genealogies in each chain as “burn-in”, we sampled every 20 genealogies for both the 10 short chains (1000 total genealogies sampled), and the three long chains (10000 total genealogies sampled). We set the transition/transversion ratio at 2.0; other parameters were left at their default settings.

To test for bias in the parameter estimates we investigated whether any mean value of θ differed from the mean values for other scenarios within a given simulated value of θ. Statistical analyses were performed using SAS version 9.0 (SAS Institute Inc., Cary, NC 2002). We used the Tukey multiple comparison adjustment to test all pairwise combinations [32].

## Results

### Number of loci

**Figure 1:**
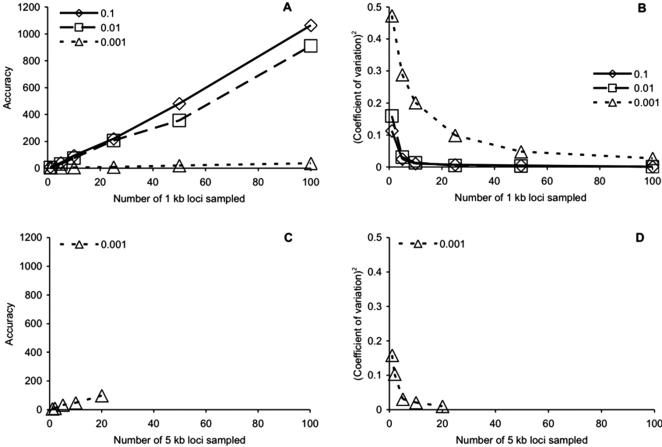
Influence of increasing the number of loci sampled per individual on the coalescent estimates of θ (0.1, 0.01, 0.001): (A) improvement in accuracy (mean^2^/variance), loci sampled are 1 kb in length; (B) improvement in squared coefficient of variation ((standard deviation/mean)^2^), loci sampled are 1 kb in length; (C) accuracy, loci sampled are 5 kb in length, θ = 0.001 (see text); (D) squared coefficient of variation, loci are 5 kb in length (see text).

As predicted by theory [14], [17] and demonstrated in previous analyses of simulated data [31], [33], estimates of θ were greatly improved by increasing the number of loci sequenced per individual (Figure 1). The accuracy, measured as the square of the mean estimate divided by the variance of the estimate, increased proportionately with the number of sampled loci (Figure 1A) and concomitantly, the square of the coefficient of variation (standard deviation/mean) decreased considerably over the same sampling regime (Figure 1B). For a given value of θ, sampling additional loci always increased the accuracy and decreased the coefficient of variation (Table 1). Further, our calculations of accuracy were largely congruent with theoretical predictions of accuracy calculated using Felsenstein's [17] modification of formulas developed by Fu and Li [34]. Discrepancies between our calculated accuracies and the predicted accuracy values arose when Migrate produced biased estimates of θ (see below)

In terms of further decreasing the squared coefficient of variation, little additional improvement was gained by sampling more than 25 loci (Figure 1B). For example, when θ = 0.01, the total improvement in the coalescent estimate, measured by subtracting the squared coefficient of variation for the 100 loci scenario (0.00111) from the squared coefficient of variation for the 1 locus scenario (0.15965) was 0.15854. Roughly 81% of the improvement was accounted for by increasing the number of loci sampled from 1 to 5, and nearly 98% of the improvement could be explained by increasing the number of sampled loci from 1 to 25; adding the last 75 loci accounted for less than 3% of the total improvement in the squared coefficient of variation. As in accuracy (above), the decrease in the squared coefficients of variation was as predicted by theory [17]. Similar results were obtained when θ = 0.1. Approximately 78% of the total improvement could be explained by increasing the number of sampled loci from 1 to 5 and nearly 97% was accounted for by increasing the number of loci sampled from 1 to 25.

Although increasing the number of sampled loci improved accuracy and decreased the squared coefficient of variation when θ = 0.001, the means of the estimates decreased (see below), precluding calculations of the improvement gained by sampling additional loci.

### Number of base pairs per locus

**Figure 2:**
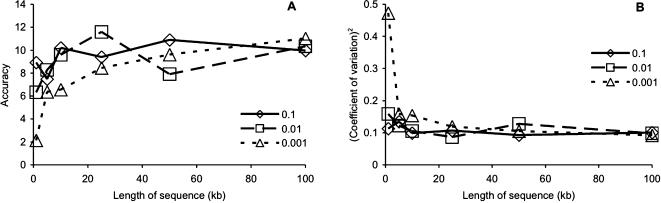
Influence on increasing the sequence length of a single sampled locus on (A) accuracy (mean^2^/variance) of the coalescent estimates of θ (0.1, 0.01, 0.001); (B) squared coefficient of variation ((standard deviation/mean)^2^).

In contrast to increasing the number of loci sampled, increasing the length of sequence at a particular locus had relatively little impact on improving the accuracy associated with estimating θ, except when the known value of θ was small (Figure 2). Increasing the length of sequence from 0.5 kb to 100 kb resulted in a total improvement in the estimate accuracy of 5.2824, when θ = 0.01, which is less than 0.6% of the total improvement in accuracy gained by increasing the number of 1 kb loci from one to 100. More dramatically, when the known value of θ was 0.001, point estimates calculated from 1 kb of sequence from a single locus were anywhere from 1.71 (min/0.001×100) to 291.3% (max/0.001×100) of the actual value (Table 1). These results demonstrate that parameter estimates based on single-locus population genetic data have large deviations regardless of sequence length or true value of the parameter of interest.

On average, across 100 replicate datasets, Migrate performed well at recovering the known value of θ (Table 1). However, Migrate produced biased estimates of θ when the known parameter value was small (0.001). The mean values of θ under some scenarios were significantly less than the true value of 0.001 (one-way ANOVA, *P*<0.05, Table 1). The downward bias resulted from the lack of information (i.e. polymorphic sites) available when the known value of θ was small (0.001 here), especially when the length of the sequence was less than 1 kb. With little information at each locus, the likelihood surfaces were very flat, leading to poor estimates of θ [31]. We addressed this problem by increasing the per-locus sequence length to 5 kb (Figures 1C and 1D), such that we generated additional datasets, which had 25 kb of sequence divided among five loci of 5 kb each, 50 kb divided equally among 10 loci, and 100 kb divided equally among 20 loci. Increasing the per-locus sequence length eliminated the downward bias (Table 1), and also increased the accuracy of the parameter calculations when compared to the values obtained for shorter sequence lengths (contrast Figures 1A and 1B with Figures 1C and 1D). Since the simulations were not extended to include datasets containing 50 and 100 loci, each 5 kb in length for θ = 0.001, we did not investigate the improvement gained by adding loci.

## Discussion

Our simulations demonstrated clearly the utility of multi-locus datasets in estimating population genetic parameters under a coalescent framework, validating a key component of theoretical investigations of optimal sampling strategies [14], [17]. An important consideration is that there are two sources of error that contribute to the variance in coalescent-based parameter estimates, error associated with calculating the probability of the genealogy and error associated with calculating the probability of the coalescent. Migrate and other coalescent-based software packages attempt to marginalize the effects of the genealogical error.

The number of loci is crucial to reducing the coefficient of variation, even more so than increasing the length of the sequence at any one locus (Figures 1, 2). In our data, the most reliable way to reduce the variation in the parameter estimates was to increase the number of loci sequenced per individual. Previous work has mentioned this issue [e.g. 11], but our results provide additional insights into how the precision of parameter estimation varies with the amount of genetic information available.

Other investigations into the optimal sampling strategy for coalescent-based estimates of population genetic parameters have used a fixed-cost approach [14], [17]. In general, they sought to identify the ratio of individuals, number of loci, and per-locus sequence length that would maximize the accuracy of the estimate. Since they operated under a fixed-cost model, there are trade-offs; for example, an increase in the number of individuals sampled necessitated a decrease the in the number of loci sampled. One important conclusion of the pioneering theoretical work by Pluzhnikov and Donnelly [14] was that to increase precision one should always choose to move to an independent region rather than extend the length of the current region. Felsenstein [17] came to similar conclusions, and described the optimal sampling strategy under a cost-per-base model as one in which large numbers of loci, each a single base long, are employed. A more realistic cost-per-read model still advocates a many-locus, few individual design, a result supported by our simulations. However, our simulations focus attention on an important caveat to the many-locus sampling strategy – the loss of information at short loci (0.5 kb in our simulations) when θ is small (0.001 in our simulations).

Under small values of θ (0.001 here), which are commonly observed in empirical data [22], [35]–[37], the paucity of informative sites in short sequences may introduce a downward bias in the estimates (Table 1). Examining the results from the simulated datasets where the total sequence data from each individual was 10 kb reveals the magnitude of this problem. At large and moderate values of θ (0.1 and 0.01), the accuracy of the estimates was highest when the 10 kb of sequence was partitioned into 20 loci, each 0.5 kb in length (Table 1). In contrast, the most accurate sampling strategy when θ = 0.001 was to sample only two loci, each 5 kb in length (Table 1). Further, when θ was 0.001, the mean value of the estimates was 0.00039 when the data were partitioned into 20 equal-length loci. This low estimate represents a statistically significant reduction of ∼60% from the known value (Table 1). Note that theoretical predictions encourage sampling more loci even at small values of θ; a 20 loci sampling strategy produced the highest predicted accuracy for each value of θ when each individual was sequenced at 10 kb. At our smallest simulated value of θ (0.001), the mean estimates of seven of the 18 datasets were significantly smaller than the known value, a potential problem with the “more loci, shorter sequence” sample design. The sampling scenarios that resulted in a biased estimate of θ were the same scenarios for which our calculated accuracy values differ greatly from the predicted values (Table 1). The predicted values are based on an unbiased coalescent estimator, which our data suggest is not the case when θ was small. The downward bias in the coalescent estimates likely accounts for the differences between our calculated accuracies and the predicted values. Increasing the sequence length at each particular locus may alleviate the problem. In our simulations, none of the datasets that contained loci 5 kb in length or longer showed the downward bias (Table 1). Further work is needed to investigate the minimum amount of sequence data per locus required to ameliorate the problem. The problem of a downward bias is well documented [17], [31], but it has not been emphasized in previous work of optimal sampling strategies. The results of our study underscore the need to consider both the per locus sequence length and number of sampled loci.

Accurate estimation of θ required data from at least 25 independently evolving loci. Beyond this, there was little added benefit in terms of decreasing the squared coefficient of variation of the coalescent estimates relative to the extra effort required to sample more loci. Interestingly, a recent paper concluded that to accurately recover phylogenetic relationships with maximum support, researchers should use at least 20 genes [38], suggesting that 20 loci will not only provide a robust estimate of population genetic parameters, but also of phylogeny. While sampling 25 loci will increase the statistical confidence in population genetic parameters, researchers interested in comparing parameter estimates between taxa should remain cautious in attributing biological significance to statistical differences [39]. These differences might be small enough to render any corresponding biological differences irrelevant.

Our analyses were based on a large, panmictic population at equilibrium, and without recombination or selection. Application of our results to empirical data must be considered in light of the restrictive assumptions under which the data were simulated. Multi-locus empirical datasets have many more sources of variation that would elevate the number of loci needed to estimate θ accurately. Another important source of variation not considered here were the theoretical limits of current coalescent methods to correctly infer population processes [40].

The calculations based on the simulated datasets were focused on generating average, multi-locus estimates of θ. Evolutionary forces (e.g. different mutation rates, levels of selection) whose impacts are heterogeneous across the genome further complicate the interpretation of empirical estimates of θ measured from loci distributed across the genome [41], [42]. For example, genetic diversity and recombination are positively correlated; genomic regions with high rates of recombination often show high nucleotide diversity [43]–[45] and vice-versa [46], [47], where selection typically reduces variation. Therefore, differences in genome-wide recombination rates [48], [49] could inflate levels of variation at some loci, beyond what might be expected for neutrally evolving loci as were sampled in our simulations.
